# Giant anterior communicating artery aneurysm with intrasellar extension

**DOI:** 10.1016/j.bas.2023.101792

**Published:** 2023-08-16

**Authors:** Arunkumar Sekar, Kavin Bharati, Vipin Chandran, Ashis Patnaik

**Affiliations:** Department of Neurosurgery, All India Institute of Medical Sciences, Bhubaneswar, Bhubaneswar, Odisha, 751019, India

**Keywords:** Panhypopituitarism, Giant intracranial aneurysm, Anterior communicating artery aneurysm, Acom aneurysm

## Abstract

**Introduction:**

Aneurysms extending into the sella are uncommon with only a few cases reported till date. Most of these arise from either the supraclinoidal or infraclinoidal segments of the internal carotid artery.

**Research question:**

Can Anterior communication artery aneurysm present with hypopituitarism due to compression of pituitary gland?

**Materials & methods:**

Case report and literature review.

**Results:**

We discuss this rare presentation in a middle-aged patient its surgical management and the follow-up course with a review of available literature.

**Discussion & conclusion:**

Anterior communicating artery aneurysms extending into the sella are extremely uncommon with only 4 cases reported in literature. They are usually giant aneurysms which are partially thrombosed with presenting with predominantly with mass effect in this case visual impairment and hypofunction of the pituitary.

## Highlights

• Aneurysms extending into the sella are uncommon and mostly from the internal carotid artery segments.
• Anterior communicating artery aneurysm extending into the sella are rare.
• They are giant aneurysm usually with partial or complete thrombosis.
• They present with mass effect to surrounding structures, in this scenario with pituitary dysfunction and visual impairment.
• Surgical management of these rare aneurysms are challenging and needs planning.

## 1 Introduction

Aneurysms projecting into the Sella turcica accounts for 1% of all aneurysms (Hanak et al., 2012; Heshmati et al., 2001). Most of it arises from either the infraclinoidal or supraclinioidal segment of internal carotid artery. Aneurysms with such intrasellar extensions presenting as hypopituitarism is extremely uncommon (Hanak et al., 2012). Of the 40 odd cases in literature only four aneurysms originate from the anterior communicating artery [ACom A] with intrasellar extension and pituitary dysfunction as a primary complaint (Hanak et al., 2012). In the current report we present the clinical course and management of one such case encountered in our practice.

## 2 Case report

**Fig. 1:**
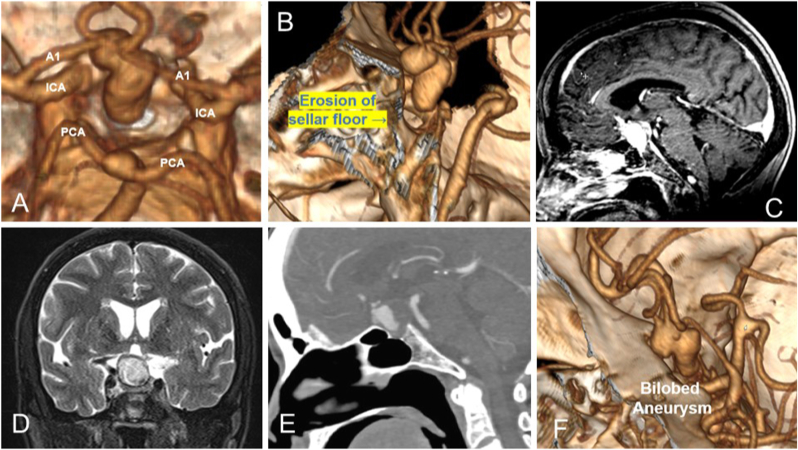
A- 3D reconstruction of aneurysm extending into the sella with vessels of circle of willis. B- 3D reconstruction of angiogram at the midsagittal level with aneurysm extending into the sella. C- T1 post contrast MRI showing enhancing mass in the sella. D- T2 hyperintense mass in the sella. E− CT angiogram showing sellar floor defect. F- bilobed morphology of the aneurysm extending into the sella.

A middle-aged patient was initially evaluated under internal medicine for complaints of mild low-grade fever and altered sensorium for one week duration. The patient hailed from a locality in which scrub typhus was endemic. There was history of poor oral intake and persistent headache. The blood investigations revealed hyponatremia. The patient was empirically started on treatment for pyrexia of unknown origin and the sodium levels were corrected. Once the sensorium improved, patient complained of blurring of vision and persisting headache. An initial brain scan revealed a lesion in sellar region with subfrontal extension with evidence of suggestive of a pituitary apoplexy. Hormonal evaluation revealed low serum cortisol levels with hypothyroidism. A contrast enhanced magnetic resonance imaging showed a lesion with T1 hypointense and T2 hypointense with areas of iso to hyper intensity with contrast enhancement in the sellar region pushing the pituitary stalk behind and compressing the normal pituitary inferiorly. On further evaluation with an angiogram revealed an inferiorly directed partially thrombosed bilobed giant aneurysm of Anterior communicating artery with erosion of sellar floor. Aneurysm was involving the origin of left A2 with dominant left A1 [Fig. 1].

**Fig. 2:**
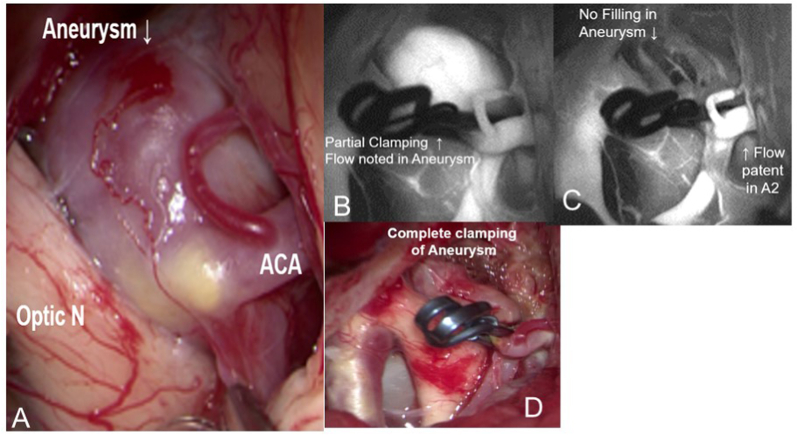
A. Intraoperative view of the giant aneurysm in relation to optic nerve and other structures. B. Indocyanine green dye used intraoperatively to identify complete clipping of aneurysm. C. After readjusting the clip and confirming exclusion of aneurysm and patent distal flow of parent vessels. D. After opening and decompression of the aneurysm dome. (For interpretation of the references to colour in this figure legend, the reader is referred to the Web version of this article.)

The patient underwent craniotomy and clip ligation of aneurysm [Fig. 2]. Intraoperative Indocyanine green [ICG] angiography showed good filling of Right and Left A1, A2 and ACom with complete exclusion of the aneurysm. Dome of the aneurysm was opened, and clots were evacuated. Both optic nerves were found to be free after decompression of clots.

Postoperatively patient had temporary diabetes insipidus which was managed appropriately. Patient recovered postoperatively without any neurological deficits and was continued on hormone replacement at 6-month follow-up.

## 3 Discussion

Giant aneurysms comprise of 5% of all intracranial aneurysms (Dengler et al., 2016). More than 50% patients present with signs and symptoms of aneurysm rupture (Lonjon et al., 2015). Giant aneurysms arising from Anterior communicating artery are rare, because of the highest risk of early rupture. The most common presentations of Giant ACom artery aneurysms are subarachnoid hemorrhage, dementia, and visual field deficits (Çağavi et al., 2006). The incidence of complete thrombosis of giant aneurysms is 3%. The incidence of partial thrombosis ranges between 27% and 74%. Thrombosis in giant aneurysms of the ACom artery has been reported about 78% (Rosta et al., 1988).

Aneurysm extending into the sella are rare but extremely eventful diagnosis as a failure to diagnose such conditions will have fatal outcome. Most of them are unruptured aneurysms presenting with mass effect on the surrounding structures (Heshmati et al., 2001; Rosta et al., 1988). Aneurysms with intrasellar extension are divided into two groups: Infradiaphragmatic aneurysms, arising from infraclinoidal segment of Internal carotid artery (Hanak et al., 2012). These aneurysms extend medially from the cavernous part of the internal carotid artery causing endocrinopathy as the predominant complaint. Supradiaphragmatic aneurysms arise from supraclinoidal segment of internal carotid or from the anterior communicating artery (Lonjon et al., 2015). Supradiaphragmatic types are usually giant aneurysms and present with visual complaints and can be partially thrombosed (Hanak et al., 2012).

The largest series available in literature is a report from Heshmati et al. (2001) who presented 7 case of aneurysms with intrasellar extension in their archival search of 50 years. Of the 4087 cases of hypopituitarism, seven cases all of them middle aged presented due to an intrasellar aneurysm. They reported a prevalence of 0.17%. Of these 7 cases, only one case was arising from anterior communicating artery. The patient had hormonal deficiency and visual impairment. The diagnosis was done intraoperatively, and patient underwent direct decompression of the aneurysm and packing with thrombogenic material. The patient was continued postoperatively on hormone replacement (Heshmati et al., 2001).

One of the reports by Aoki et al. (Aoki, 1988), mentions about a patient who presented with sudden onset of visual deterioration following sentinel headaches a few days earlier. His imaging showed a sellar lesion which was initially suspected to be pituitary apoplexy. The angiogram revealed a giant aneurysm of ACom artery partially thrombosed and extending into the sella. The patient underwent clip ligation and decompression of the aneurysm. Postop he had transient diabetes insipidus but had residual visual field deficits at 3-month followup (Aoki, 1988).

Gilad et al. (2007), presented a rare clinical scenario in which a middle age gentleman with hypertension presented with sudden onset of occipital headache and back pain which gradually progressed to headache, nausea and diplopia. Imaging revealed a subtentorial acute subdural hemorrhage without any evidence of subarachnoid hemorrhage. Further evaluation showed aneurysm of the ACom artery with extension into the sella with rupture into the sella. The aneurysm managed by endovascular coiling (Gilad et al., 2007).

Murai et al. (2004), presented a patient who was evaluated for headache and a sellar mass identified by magnetic resonance imaging. Angiography revealed ACom artery aneurysm directed into the sella. However during surgery due to technical difficulties, the procedure was abandoned and patient was kept on a close followup (Murai et al., 2004).

The most recent report from zhao et al. (Zhao et al., 2015), presented a middle-aged lady with multiple sentinel headaches and irregular menstrual periods was initially managed medically and later found on routine imaging to have a sellar lesion. Her hormone profile was normal. Angiography revealed an ACom artery aneurysm projecting into the Sella. She underwent clip ligation of the aneurysm. At 6-month followup she had improved clinically with resumption of regular menstrual cycles (Zhao et al., 2015).

Surgical Clipping for giant intracranial aneurysm is complex than conventional clipping of smaller aneurysms. Giant aneurysms frequently requires temporary clipping or trapping of the parent vessel, which makes neuromonitoring and neuroprotection techniques mandatory. Microsurgical bypass is indicated in cases of planned or unplanned sacrifice of the parent artery to prevent long-term ischemic complications (Zhao et al., 2015). Although there are a few cases of intrasellar aneurysms which were treated with endovascular coiling, when it comes to symptoms relief in the form of visual impairment and hypopituitarism using advanced endovascular techniques the literature is limited (Hanak et al., 2012).

## 4 Conclusion

Panhypopituitarism in the background of Giant intracranial aneurysms are rare entity. In the presence of giant ACom artery aneurysm extending into sella, preop diagnosis is difficult and the presence of thrombus within the aneurysm make the diagnosis even more difficult. Angiography plays a role in diagnosing the condition and also helps in planning a specific treatment. There is limited available literature about endovascular management of these subset of aneurysms, especially when it comes to relief of compressive symptoms (Hanak et al., 2012). Micro neurosurgical techniques play a major role for giant aneurysms with intrasellar extension with visual pathway compression and hypopituitarism as it can reliably ligate the aneurysm and relief of compressive symptoms (Hanak et al., 2012).

## Informed consent

Written informed consent was obtained from the patient for publication of this case report and accompanying images.

## Author contributions

Dr Arunkumar Sekar-manuscript preparation, data collection and patient care. Dr. Kavin Bharati - manuscript preparation and data collection. Dr Vipin Chandran and Dr Rabi Narayan Sahu were involved in patient care and review.

## Financial support

The authors did not receive support from any organization for the submitted work.

## Declaration of competing interest

There are no conflicts of interest for any of the authors involved in the paper.
