# Supplementary material for: Giant anterior communicating artery aneurysm with intrasellar extension
Source: Brain Spine. 2023 Aug 16;3:101792. doi: 10.1016/j.bas.2023.101792 (PMC10668054; doi:10.1016/j.bas.2023.101792)
Supplement: Multimedia component 1 [file mmc1.docx]

**Structured Review:**

The largest series available in literature is a report from Heshmati et al[3] who presented 7 case of aneurysms with intrasellar extension in their archival search of 50 years. Of the 4087 cases of hypopituitarism, seven cases all of them middle aged presented due to an intrasellar aneurysm. They reported a prevalence of 0.17%. Of these 7 cases, only one case was arising from anterior communicating artery. The patient had hormonal deficiency and visual impairment. The diagnosis was done intraoperatively, and patient underwent direct decompression of the aneurysm and packing with thrombogenic material. The patient was continued postoperatively on hormone replacement.

One of the reports by Akoi et al[4], mentions about a patient who presented with sudden onset of visual deterioration following sentinel headaches a few days earlier. His imaging showed a sellar lesion which was initially suspected to be pituitary apoplexy. The angiogram revealed a giant aneurysm of ACom artery partially thrombosed and extending into the sella. The patient underwent clip ligation and decompression of the aneurysm. Postop he had transient diabetes insipidus but had residual visual field deficits at 3-month followup.

Gilad et al,[5] presented a rare clinical scenario in which a middle age gentleman with hypertension presented with sudden onset of occipital headache and back pain which gradually progressed to headache, nausea and diplopia. Imaging revealed a subtentorial acute subdural hemorrhage without any evidence of subarachnoid hemorrhage. Further evaluation showed aneurysm of the ACom artery with extension into the sella with rupture into the sella. The aneurysm managed by endovascular coiling.

Murai et al,[6] presented a patient who was evaluated for headache and a sellar mass identified by magnetic resonance imaging. Angiography revealed ACom artery aneurysm directed into the sella. However during surgery due to technical difficulties, the procedure was abandoned and patient was kept on a close followup.

The most recent report from zhao et al,[7] presented a middle-aged lady with multiple sentinel headaches and irregular menstrual periods was initially managed medically and later found on routine imaging to have a sellar lesion. Her hormone profile was normal. Angiography revealed an ACom artery aneurysm projecting into the Sella. She underwent clip ligation of the aneurysm. At 6-month followup she had improved clinically with resumption of regular menstrual cycles.

Surgical Clipping for giant intracranial aneurysms are complex than conventional clipping of smaller aneurysms. Giant aneurysms frequently requires temporary clipping or trapping of the parent vessel, which makes neuromonitoring and neuroprotection techniques mandatory. Microsurgical bypass is indicated in cases of planned or unplanned sacrifice of the parent artery to prevent long-term ischemic complications[7].
